# BOLD signal variability and complexity in children and adolescents with and without autism spectrum disorder

**DOI:** 10.1016/j.dcn.2019.100630

**Published:** 2019-03-05

**Authors:** Amanda K. Easson, Anthony R. McIntosh

**Affiliations:** a Rotman Research Institute, Baycrest Hospital, 3560 Bathurst Street, Toronto, ON, M6A 2E1, Canada; b Department of Psychology, University of Toronto, 100 St. George Street, Toronto, ON, M5S 3G3, Canada

**Keywords:** ABIDE, autism brain imaging data exchange, ADHD, attention deficit hyperactivity disorder, ADOS2, autism diagnostic observation schedule 2, ASD, autism spectrum disorder, BSR, bootstrap ratio, FC, functional connectivity, DWI, diffusion weighted imaging, GE, global efficiency, MSSD, mean square successive difference, PLS, partial least squares, RRB, restricted and repetitive behaviour, SA, social affect, SC, structural connectivity, SRS, social responsiveness scale, TD, typically developing, Brain-behavior relationships, Mean square successive difference, Partial least squares, Resting-state fMRI, Sample entropy

## Abstract

Variability of neural signaling is an important index of healthy brain functioning, as is signal complexity, which relates to information processing capacity. Alterations in variability and complexity may underlie certain brain dysfunctions. Here, resting-state fMRI was used to examine variability and complexity in children and adolescents with and without autism spectrum disorder (ASD). Variability was measured using the mean square successive difference (MSSD) of the time series, and complexity was assessed using sample entropy. A categorical approach was implemented to determine if the brain measures differed between diagnostic groups (ASD and controls). A dimensional approach was used to examine the continuum of relationships between each brain measure and behavioral severity, age, IQ, and the global efficiency (GE) of each participant’s structural connectome, which reflects the structural capacity for information processing. Using the categorical approach, no significant group differences were found for neither MSSD nor entropy. The dimensional approach revealed significant positive correlations between each brain measure, GE, and age. Negative correlations were observed between each brain measure and the severity of ASD behaviors across all participants. These results reveal the nature of variability and complexity of BOLD signals in children and adolescents with and without ASD.

## Highlights

• Resting-state BOLD signal variability and complexity were examined.
• No significant group differences were observed in youth with and without autism.
• A continuum of brain-behavior relationships was observed across diagnostic groups.
• Positive correlations were found between brain measures, age and global efficiency.
• Negative correlations were found between the brain measures and behavioral severity.

## 1 Introduction

Two key components of healthy brain functioning are variability of neural signaling and complexity of these signals. In the healthy brain, variability of neural signaling allows for the formation of functional networks (Fuchs et al., 2007) and the exploration of multiple stable functional states (Ghosh et al., 2008; McIntosh et al., 2008, 2010). Previous work has shown that higher variability of brain signals is associated with better behavioral performance (Garrett et al., 2011). Further, variability changes in response to task demands: BOLD variability has been shown to be lowest in the resting-state, increased during internally focused tasks, and highest during externally focused tasks (Grady and Garrett, 2018). It has been suggested that variability allows for greater environmental uncertainty during externally-directed compared to internally-directed tasks, and variable, flexible neural signaling allows the brain to adapt to such uncertainty (Grady and Garrett, 2018).

Several studies have examined age related changes in brain signal variability. Using fMRI, Garrett et al. (2010) found that during fixation periods of a task, the standard deviation of BOLD time series increased with age in 33% of voxels and decreased in 67% of voxels. The spatial pattern of age-related changes in variability was different than that of mean BOLD activity, suggesting that these metrics provide unique information about age-related changes in brain activity. Variability has also been examined using the mean square successive difference (MSSD; von Neumann et al., 1941), defined as the average of the sum of squared differences in amplitude between successive time points. Thus, MSSD measures variability from one time point to the next. MSSD reflects variability in a signal that is independent of drifts in the mean (Garrett et al., 2011). Using fMRI, Nomi et al. (2017) found that MSSD decreased from ages 6 to 85 in the majority of brain regions examined. In attention deficit hyperactivity disorder (ADHD), positive correlations have been reported between symptom severity and MSSD in default mode regions (Nomi et al., 2018).

Signal complexity is also important for optimal brain function. One way to measure the complexity of a signal is with sample entropy, which assigns high values to more complex signals, and low values to highly deterministic or random signals (Costa et al., 2005). It is important to measure the complexity of brain signals in addition to variability, because a variable brain signal may not necessarily be complex. A signal with higher entropy can be interpreted as having higher information processing capacity (Gatlin, 1972; Heisz and McIntosh, 2013; Shannon, 1948). Like variability, complexity is thought to reflect the ability of the brain to adapt to unpredictable environments (Goldberger et al., 2002). Using fMRI, it has been shown that signal complexity in various regions of the default mode network was positively correlated with multiple cognitive functions, including attention, language, and short-term memory (Yang et al., 2013). Higher multiscale entropy of EEG signals has been associated with greater knowledge representation (Heisz et al., 2012). It has further been shown that entropy of EEG signals increases across development (Lippé et al., 2009; McIntosh et al., 2008; Misic et al., 2010).

Abnormal levels of variability and complexity in the brain may be related to sub-optimal cognition. Too much variability can result in inefficient information processing and ineffective exploration of different network configurations in the brain (Ghosh et al., 2008; McIntosh et al., 2008, 2010). Autism spectrum disorder (ASD), a neurodevelopmental disorder that is characterized by atypical social communication and restricted, repetitive and stereotyped behaviors (American Psychiatric Association, 2013), is one condition that may be characterized by detrimental levels of noise. ASD is hypothesized to be characterized by an increased ratio of excitatory to inhibitory coupling in the brain, which could be associated with noisier and less stable signaling, reduced functional differentiation, and less efficient information processing (Rubenstein and Merzenich, 2003). These alterations may relate to abnormal myelination and synaptic development (Rubenstein and Merzenich, 2003). Inhibitory signaling is believed to be associated with improving the specificity of excitatory brain signals; thus, imprecise brain activity and increased noise can result from increased excitatory and decreased inhibitory signaling (Nelson and Valakh, 2015).

Studies of dynamic functional connectivity (FC) have shown that FC is also more variable in ASD (e.g. Chen et al., 2017; Falahpour et al., 2016; Wee et al., 2016; Zhang et al., 2016). Chen et al. (2017) reported greater variance of distributed functional connections in ASD, which was related to total scores on the Autism Diagnostic Observation Schedule (ADOS). Dynamic FC has been shown to improve classification of ASD compared to typically developing (TD) participants (Wee et al., 2016), and to be a better predictor of ASD behaviours compared to “static” FC measured over an entire time series (Jia et al., 2014). However, the nature of resting-state BOLD signal variability in ASD compared to TD individuals remains unclear. Entropy of EEG signals has also been shown to be reduced in ASD at rest (Bosl et al., 2011) and during both social and non-social tasks (Catarino et al., 2011), but the nature of the complexity of resting-state BOLD time series in ASD is currently unknown. Further, relationships between information processing capacity in functional compared to structural networks in ASD are not well-characterized. Anatomy plays a role in shaping functional networks in the brain (Deco et al., 2013; Honey et al., 2007, 2009, 2010). For instance, regions that exhibit high structural connectivity (SC) typically exhibit strong FC (Honey et al., 2007, 2009). Previous work has revealed relationships between information processing capacity in structural networks and cognitive functioning (Berlot et al., 2016; Reijmer et al., 2013). Diffusion weighted imaging (DWI) can be used to estimate the strength of white matter connections between brain regions via tractography. As time series are not available for structural connectomes, a graph theory metric, global efficiency (GE), can be used to estimate information processing capacity (Bullmore and Sporns, 2012).

In the present study, we characterized BOLD signal variability and complexity in youth with and without ASD. Further, we characterized relationships between these measures and age, GE, IQ, and behavioral severity.

## 2 Materials and methods

### 2.1 Participants

Twenty male participants with ASD (M = 13.25 years, SD = 2.87 years) and 17 male TD participants (M = 13.42 years, SD = 3.21 years) from the San Diego State University sample from the Autism Brain Imaging Data Exchange (ABIDE) II database (Di Martino et al., 2017) were included in this study. Informed consent or assent had been obtained for all participants and caregivers in accordance with the University of California, San Diego and San Diego State University Institutional Review Boards. Participants were matched for age, full-scale IQ and head motion (Table 1). Participants were excluded if they were less than 8 years of age, their full-scale IQ was below 75 or if their head motion during the scan exceeded a mean framewise displacement (FD) 0.2 mm. Further, participants were included only if they had an MPRAGE scan, resting-state fMRI scan, and DWI scan.

**Table 1:** Participant Characteristics.

Parameter	ASD (Mean + SD) [range]	TD (Mean + SD) [range]	Group difference
N	20	17	N/A
Age	13.25 + 2.87 [9.6 – 17.80]	13.42 + 3.21 [8.10 – 17.60]	*t*(35) = -0.17, *p* = 0.87
IQ	98.50 + 14.33 [77 – 130]	103.35 + 10.15 [79 – 125]	*t*(35) = -1.17, *p* = 0.25
Handedness	15 RH 3 LH 2 mixed	14 RH 1 LH 2 mixed	*X*^2^(2, *N* = 37) = 0.80, *p* = 0.67
Head motion: fMRI (mean FD)	0.106 + 0.035 [0.054 – 0.189]	0.096 + 0.042 [0.040 – 0.194]	*t*(35) = 0.76, *p* = 0.45
Head motion: fMRI (number of censored time point)	3.85 + 1.69 [1 – 7]	3.35 + 1.84 [0 – 7]	*t*(35) = 0.86, *p* = 0.40
Head motion: DWI (mean FD)	0.609 + 0.155 [0.426 – 0.999]	0.546 + 0.160 [0.325 – 1.05]	*t*(35) = 1.21, *p* = 0.24
SRS Total	104.25 + 24.92 [59 – 147]	18.41 + 11.57 [2 – 36]	*t*(35) = 13.04, *p* < 0.001
SRS Awareness	13.10 + 4.39 [3 – 19]	3.82 + 2.83 [0 – 10]	*t*(35) = 7.48, *p* < 0.001
SRS Cognition	17.85 + 6.02 [4 – 28]	2.94 + 2.77 [0 – 8]	*t*(35) = 9.39, *p* < 0.001
SRS Communication	34.95 + 10.36 [21 – 55]	5.88 + 4.48 [0 – 15]	*t*(35) = 10.72, *p* < 0.001
SRS Motivation	17.80 + 4.12 [11 – 25]	3.94 + 2.44 [0 – 8]	*t*(35) = 12.15, *p* < 0.001
SRS Mannerisms	22.05 + 7.86 [8 – 36]	1.82 + 2.77 [0 – 11]	*t*(35) = 10.08, *p* < 0.001
ADOS2 Total	14.70 + 4.92 [5 – 24]	N/A	N/A
ADOS2 SA	11.35 + 4.26 [5 – 20]	N/A	N/A
ADOS2 RRB	3.35 + 1.95 [0 – 8]	N/A	N/A
ADOS2 Severity	7.85 + 2.08 [3 – 10]	N/A	N/A

### 2.2 Data acquisition

The following scans were acquired on a GE MR750 system at SDSU: structural (T1-weighted 3D SPGR sequence; TR = 8.136 ms, TE = 3.172 ms, TI = 600 ms, flip angle = 8°, field of view (FOV) = 256 mm, matrix size 256 × 192, 1.0 mm isotropic voxel resolution), diffusion-weighted (T2-weighted sequence; TR = 8500 ms, FOV = 192 mm, matrix size 96 × 96, in-plane voxel dimension of 0.9375 × 0.9375 mm/1.875 × 1.875 mm with 2 mm slice thickness, 68 slices, 61 diffusion directions), and fMRI (two-dimensional T2-weighted gradient echo planar imaging blood oxygen level-dependent contrast sequence; TR = 2000 ms, TE = 30 ms, flip angle = 90°, FOV = 200 mm, in-plane voxel dimension of 3.4375 mm x 3.4375 mm voxel resolution, 3.4 mm slice thickness, matrix size 64 × 64, 42 slices, 180 TRs, eyes open).

### 2.3 Resting-state fMRI preprocessing

Data were preprocessed using the Optimization of Preprocessing Pipelines for NeuroImaging (OPPNI) software (Churchill et al., 2012a,b, 2015). First, motion correction was performed using AFNI’s 3dvolreg function, followed by the generation of subject-specific non-neuronal tissue masks using the PHYCAA + algorithm (Churchill and Strother, 2013), then replacement of outlier volumes with interpolated values from neighbouring time points (Campbell et al. (2013); https://www.nitrc.org/projects/spikecor_fmri). This motion censoring step was implemented due to previous evidence suggesting that censoring is highly effective at removing residual motion-related artifacts in resting-state fMRI data. For instance, Ciric et al. (2017) noted that censoring mitigates motion artifacts as well as distance-dependent artifacts. They also found that methods that are less effective at denoising are not able to identify modular network structure in functional connectomes. They noted that global signal regression can mitigate motion artifacts, but has the drawback of introducing distance-dependent artifacts. Thus, in this study, censoring was used to remove residual motion artifacts. Importantly, the maximum number of time points that were censored was 7 out of 175, and the number of censored time points did not differ between ASD and TD groups. Nonetheless, we analyzed the effects of censoring on MSSD and entropy estimates. These analyses are described in the Supplementary Material.

These steps were followed by slice-timing correction and temporal detrending using a second-order Legendre polynomial. Next, principal component analysis was performed on the motion parameters obtained from the motion correction step. Principal components that accounted for more than 85% of the variance of the motion parameters were regressed out of the fMRI data. The time series of the mean white matter and CSF signals were regressed out of the data, and finally, lowpass filtering was performed with a cutoff of 0.1 Hz.

**Fig. 1:**
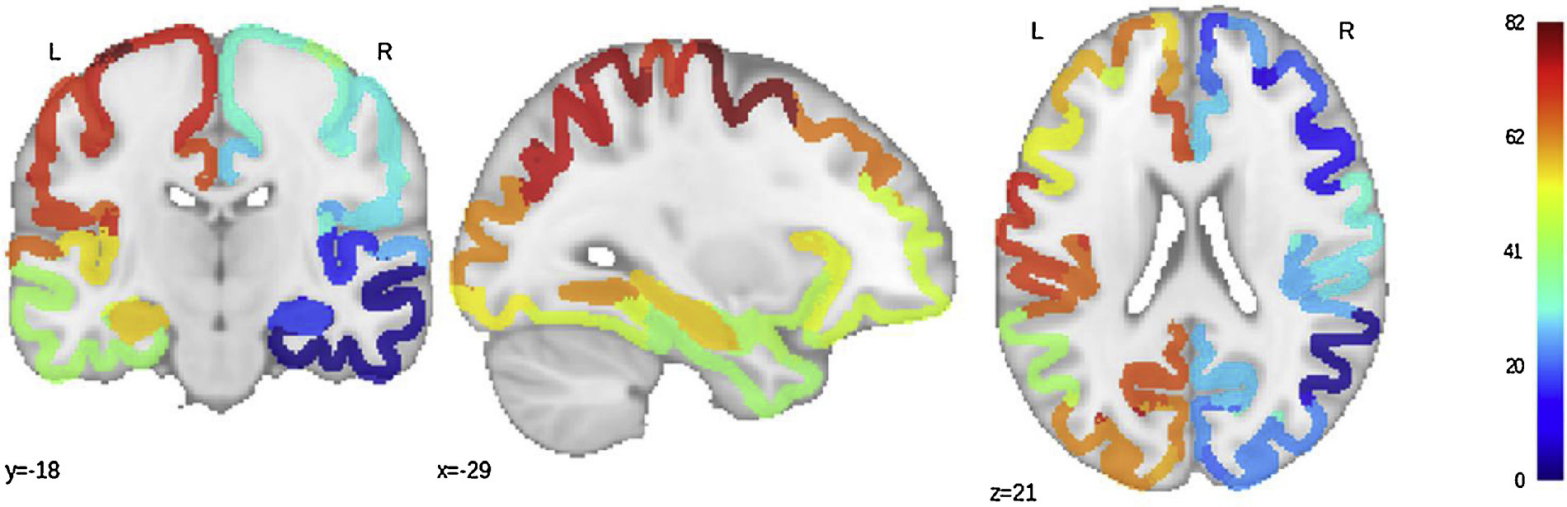
Cortical regions from the Bezgin et al. (2012) atlas in MNI space.

The ROI atlas used in this study is described in Bezgin et al. (2012). Parcellation of each hemisphere into 48 regions was performed on a macaque brain surface using mapping rules established by Kötter and Wanke (2005). The parcellation was then transformed to the MNI human brain using landmarks from Caret (www.nitrc.org/projects/caret; Van Essen et al., 2001; Van Essen and Dierker, 2007). As direct anatomical connections have been measured in the macaque brain, using this parcellation scheme helps to eliminate false positive connections found by probabilistic tractography used in human DWI analyses. This atlas is the default atlas used in *The Virtual Brain* (Sanz Leon et al., 2013) to simulate brain network dynamics from human neuroimaging data. Further, as noted in Bezgin et al. (2012), “although the detailed anatomy of the human brain is still poorly understood, there exist many homologies with the brains of non-human primates. Thus, a viable route to enhance the functional-anatomical framework in humans would be to map human brain regions to areas that have been well studied in invasive monkey experiments”. For this study, we only used cortical regions from the atlas, resulting in 82 ROIs in total (41 per hemisphere; Table 2; Fig. 1). The atlas was transformed from MNI space to each subject’s T1 space, then subsequently transformed to each subject’s functional space using the inverse of each subject’s functional-to-anatomical transform, which was obtained using linear registration with 6 ° of freedom (DOF). Next, the time series of each region was extracted using the mri_segstats function in Freesurfer.

**Table 2:** Cortical regions from the Bezgin et al. (2012) atlas.

Index		Region
Right	Left	
1	42	Temporal polar cortex
2	43	Superior temporal cortex
3	44	Amygdala
4	45	Orbitoinferior prefrontal cortex
5	46	Anterior insula
6	47	Orbitomedial prefrontal cortex
7	48	Central temporal cortex
8	49	Orbitolateral prefrontal cortex
9	50	Inferior temporal cortex
10	51	Parahippocampal cortex
11	52	Gustatory cortex
12	53	Ventrolateral premotor cortex
13	54	Anterior visual area, ventral part
14	55	Posterior insula
15	56	Prefrontal polar cortex
16	57	Hippocampus
17	58	Subgenual cingulate cortex
18	59	Ventrolateral prefrontal cortex
19	60	Visual area 2 (secondary visual cortex)
20	61	Medial prefrontal cortex
21	62	Ventral temporal cortex
22	63	Anterior visual area, dorsal part
23	64	Visual area 1 (primary visual cortex)
24	65	Centrolateral prefrontal cortex
25	66	Secondary auditory cortex
26	67	Retrosplenial cingulate cortex
27	68	Posterior cingulate cortex
28	69	Anterior cingulate cortex
29	70	Secondary somatosensory cortex
30	71	Primary somatosensory cortex
31	72	Primary auditory cortex
32	73	Primary motor cortex
33	74	Inferior parietal cortex
34	75	Medial parietal cortex
35	76	Dorsomedial prefrontal cortex
36	77	Intraparietal cortex
37	78	Superior parietal cortex
38	79	Frontal eye field
39	80	Dorsolateral prefrontal cortex
40	81	Medial premotor cortex
41	82	Dorsolateral premotor cortex

### 2.4 DWI preprocessing

Preprocessing of dwMRI data included motion correction using eddy current correction in FSL, fitting diffusion tensor models at each voxel using the dtifit function in FSL, co-registering each subject’s skull-stripped T1 MRI to DTI space using linear registration with 12 ° of freedom (DOF), registering a standard MNI T1 image to each subject’s T1 image, labeling the grey matter in T1 space with the 82 cortical regions using nearest neighbour interpolation, then transforming the ROI-labelled T1 image into diffusion space. BEDPOSTX (Bayesian Estimation of Diffusion Parameters Obtained using Sampling Techniques; the X refers to the modeling of crossing fibres) in FSL was used to fit the probabilistic diffusion model on the preprocessed dwMRI data. Markov Chain Monte Carlo sampling is run to build up distributions on diffusion parameters at each voxel. Probabilistic fiber tracking using the probtrackx2 function in FSL was then performed to define weights (fiber counts/number of streamlines) and anatomical distances between each pair of ROIs. Probtrackx2 takes repetitive samples from the distributions of voxel-wise principal diffusion directions. A streamline is computed through each of these local samples, thus generating a probabilistic streamline. By taking many samples, a histogram of the posterior distribution of the streamline location, or connectivity distribution, is created. All masks for tractography were interface masks, that is, masks of the boundary between the gray matter and white matter. This approach is referred to as anatomically-constrained tractography, and allows one to avoid the white matter seeding bias, that is, the tendency for major white matter structures to be over-defined (Smith et al., 2012). The seeds for tractography were masks of each of the 82 ROIs. The following settings were used: 5000 samples, 2000 steps per sample, step length of 0.5, curvature threshold of 0.2, loop check on, and correction of the path distribution for the length of the pathways. Targets were masks for each of the other 81 ROIs; streamlines were terminated once they reached the target mask. For a given ROI, the exclusion mask for connections with ipsilateral ROIs consisted of that ROI and ROIs in the contralateral hemisphere, and the exclusion mask for connections with contralateral ROIs was the mask for that ROI. Any pathways that entered the exclusion masks were discarded. For each subject, an SC weights matrix was defined for each pair of ROIs as the number of streamlines detected between the two ROIs divided by the total number of samples. A tract lengths matrix was defined for each pair of ROIs as the average distance between the two ROIs. Finally, both the weights and tract lengths matrices were thresholded based on tractography data for the same atlas from the CoCoMac database (Stephan et al., 2001), such that non-existent connections in the CoCoMac data were set to 0 in the human weights and tract lengths matrices to control for false positives.

Next, the GE of each participant’s SC weights matrix was calculated. GE was used as a summary measure of structural networks because a single value is calculated for the entire network, instead of one measure for each ROI. GE is defined as the average inverse shortest path length in the brain network, and is a measure of the overall capacity of the brain network to perform “parallel information transfer and integrated processing” (Bullmore and Sporns, 2012). Therefore, it would be expected that brain networks that exhibit high GE in their structural networks would exhibit high overall entropy of functional networks. GE was calculated for each participant’s SC matrix using the *efficiency_wei.m* function from the Brain Connectivity Toolbox (Rubinov and Sporns, 2010). We used a graph metric to estimate information processing capacity in each participant’s structural network, since time series are not available for estimating variability or entropy.

Based on previous work examining correlations between GE and age that controlled for effects of head motion (Rudie et al., 2012), we examined the correlation between GE and mean FD that was calculated for the DWI scans, and found that this relationship was significant, *r*(35) = −0.39, *p* =  0.02. Therefore, the effects of head motion were regressed out of the GE values.

### 2.5 BOLD signal variability and complexity

Prior to calculating variability and complexity, the BOLD time series for each ROI was normalized to have a mean of 0 and standard deviation of 1. BOLD signal variability was defined using MSSD (von Neumann et al., 1941), which is defined as the sum of the squared differences of BOLD signal values between successive time points for a region of interest, divided by the number of time points minus one. A primary benefit of MSSD is that it prevents overestimates of dispersion in data that result from a shift in the mean (Garrett et al., 2011). The formula for MSSD is as follows:

$$\delta^{2}=\frac{\sum_{i=1}^{n-1}{\left(x_{i+1}-x_{i}\right)}^{2}}{n-1}$$

Complexity was defined as the sample entropy of the signal using the sample entropy function from PhysioNet (https://physionet.org/physiotools/mse/). Sample entropy is used to determine the appearance of repetitive patterns in a time series, thus measuring the regularity of the time series. As such, sample entropy values are low for signals that are completely random or strongly deterministic, and are high for more complex signals. (Costa et al., 2005; Pincus, 1991; Richman and Moorman, 2000).

One issue with measuring sample entropy is that two parameters must be selected: the pattern length *m*, which is the number of data points used for pattern matching, and the tolerance factor *r*, which is the fraction of the standard deviation of the signal. Recently, Yang et al. (2018) presented a method for selecting optimal m and r values for an fMRI dataset based on the standard error of entropy estimates in CSF voxels. For different combinations of m and r, entropy was calculated for each voxel in each participant’s CSF mask. Then, the standard error of this entropy estimate was defined as

$$SE=1.96\text{*}\;\frac{\left(\frac{\sigma SampEn}{SampEn}\right)}{2}$$

as in Yang et al. (2018). The median standard error was then calculated across participants, for each combination of m and r. Further, the number of sample entropy estimation failures due to an absence of pattern matches was summed across all CSF voxels and participants. “Acceptable” combinations of m and r were defined as those with a median standard error of less than 0.1, and with zero sample entropy estimation failures across all CSF voxels and participants. These values were used to calculate the sample entropy of the ROIs in the grey matter. Subsequently, for each participant, sample entropy values were averaged across the acceptable m and r combinations for each ROI. Using this method, we tested m values of 1 to 4 with a step size of 1, and r values ranging from 0.05 to 0.80, with a step size of 0.05, which were the same r values used in Yang et al. (2018). These investigators also tested greater values of m, but found that for m > 4, the standard error was greater than 0.1 for all values of r; thus, we only test m values of 4 or less.

### 2.6 Partial least squares

Partial least squares (PLS; McIntosh et al., 1996; McIntosh and Lobaugh, 2004) is a multivariate statistical method that is used to determine optimal relationships between a set of brain variables and either a study design (mean-centering PLS) or a set of behaviour variables (behavioural PLS). In PLS, singular value decomposition is used to calculate orthogonal patterns that explain the maximal covariance between brain variables and design or behaviour variables. For each pattern, “brain saliences” are calculated for each brain region to indicate how strongly each region contributes to the relationship between brain and design/behaviour. In other words, brain saliences indicate which brain regions best characterize the relationship. In mean-centering PLS, design saliences indicate the group, condition, or group *x* condition profiles that best characterize the relationship between the brain and design variables. In the case of behavioural PLS, behaviour saliences indicate the profile of behaviour variables that best characterize the relationship between the set of brain variables and behaviour variables. “Brain scores” and “behaviour scores” are calculated for each pattern of relationships between brain and behaviour variables, which indicate the contribution of each participant’s brain and behaviour variables, respectively, to the pattern. Brain scores are calculated by multiplying the matrix of brain variables by the brain saliences; behaviour scores are calculated by multiplying the matrix of behaviour variables by the behaviour saliences. Further, singular values show the proportion of covariance that each pattern (relationship between brain and design/behaviour) accounts for.

In this study, mean-centering PLS was used to determine optimal contrasts in entropy between conditions (acceptable combinations of m and r), and optimal contrasts in MSSD and entropy between ASD and TD groups. Behavioural PLS was used to determine relationships each of these brain variables and a set of predictor variables, including GE of the structural networks, age, IQ, and scores on the Social Responsiveness Scale (SRS; Constantino and Gruber, 2005). The SRS is a parent or teacher report of ASD-related traits that was created for use in the general population in both clinical and educational settings. Behavioural PLS was also performed using scores on the Autism Diagnostic Observation Schedule 2 (ADOS2) instead of SRS scores in the ASD group. This analysis was only performed for ASD participants, as ADOS2 scores were not available for the TD group. Each behaviour variable was normalized to have a mean of 0 and standard deviation of 1.

The significance of each PLS pattern can be determined using permutation testing. The rows (participants) of the data matrix were reshuffled and the singular value was recalculated. This procedure was repeated 1000 times to obtain a distribution of singular values. Then, a p-value for the original singular value was obtained by calculating the proportion of singular values from the sampling distribution that are greater than the original singular value.

Further, the reliability of each brain salience can be determined using a bootstrapping procedure. Here, 500 bootstrap samples were generated by randomly sampling participants with replacement while maintaining group membership. Next, for each brain salience (brain region), a bootstrap ratio (BSR) was calculated as the ratio of the brain salience to the standard error of the salience from the bootstrap samples, which is a measure of the stability of each brain salience regardless of which participants are included in the analysis. Stable brain regions were defined as those that surpassed a BSR threshold of +2, which corresponds to approximately a 95% confidence interval.

### 2.7 Data visualization

Brain plots were created using the following Python packages: matplotlib (Hunter, 2007), nibabel (Brett et al., 2016), and nilearn (Abraham et al., 2014). All other figures were created in MATLAB (version R2015b).

## 3 Results

### 3.1 Effects of m and r on entropy estimates

**Fig. 2:**
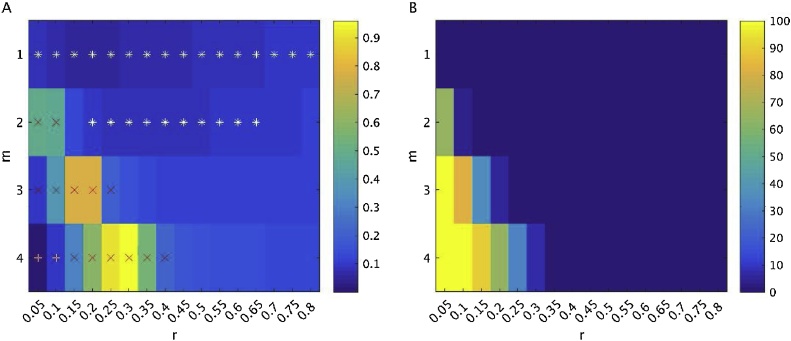
A) Standard error of entropy estimates in CSF voxels. White stars indicate “acceptable” combination of m and r, for which the median entropy estimate in CSF voxels across participants was less than 0.1. Red crosses indicate combinations of m and r that resulted in a failure of entropy estimation in at least 1 voxel across all participants. B) Percentage of all CSF voxels (across all participants) showing a failure of entropy estimation.

Fig. 2 shows the results of the entropy estimates in CSF voxels. This analysis revealed that for m = 1, all r values were acceptable. For m = 2, r values from 0.20 to 0.65 were acceptable. Hereafter, the term “condition” will be used to refer to an acceptable combination of m and r.

To ensure that any group differences in MSSD and entropy in the gray matter ROIs were not confounded by residual effects of head motion, we first performed a behavioral PLS using mean FD as the “behaviour” variable. We found that there was no significant relationship with head motion for MSSD (*p* =  0.53) or entropy (*p* =  0.23). However, we found that the relationship between SD and head motion was significant (*p* =  0.002), even when additional ICA denoising was implemented using ICA-AROMA (*p* =  0.005; Pruim et al., 2015a, 2015b). These findings therefore provide more support for the use of MSSD over SD for measuring brain signal variability.

**Fig. 3:**
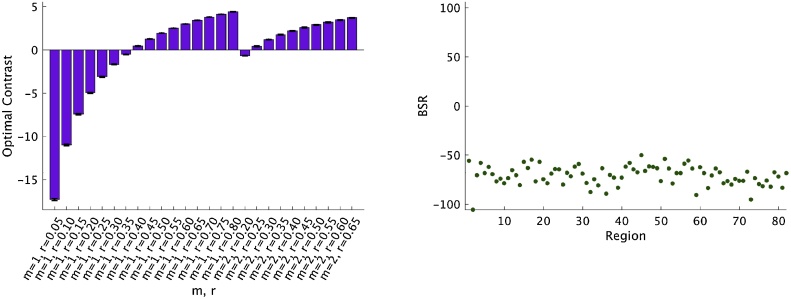
Contrast between entropy conditions and associated BSRs for each region, at a threshold of +2. Error bars show 95% confidence intervals determined through bootstrap resampling.

Mean-centering PLS was then used to examine how different acceptable combinations of m and r affected entropy estimates in the 82 grey matter ROIs across all participants. There was one significant pattern from this analysis that showed a contrast between different combinations of m and r (p < 0.001, 99.97% covariance explained; Fig. 3). Therefore, entropy estimates differ significantly depending on which combinations of m and r are used for the analyses.

For subsequent analyses, since there were differences in entropy that depended on the choice of m and r, we averaged entropy estimates across all acceptable m = 2 conditions except for r = 0.20, which showed a contrast with the other r values for m = 2.

### 3.2 Categorical analyses of MSSD and entropy using mean-centering PLS

Next, mean-centering PLS was performed to determine differences in MSSD and entropy between diagnostic groups. In other words, a categorical approach was used to analyze MSSD and entropy. For MSSD, no significant differences were observed between ASD and TD groups (*p* =  0.18). Similarly, groups were not significantly different in entropy (*p* =  0.15).

### 3.3 Dimensional analyses of MSSD and entropy using behavioral PLS

Next, behavioral PLS was performed to characterize relationships between MSSD and entropy in each brain region and the following measures: GE, age, IQ, and SRS scores, and between entropy and these measures. One main advantage of using PLS in this way is that relationships between different “behaviour” variables in relation to brain variables can be analyzed, for instance, to determine if two behaviour variables differ in terms of the strength or direction of their linear relationships with the brain variables. PLS was performed using SRS total scores as opposed to scores on the five SRS subscales, because scores on the subscales were highly correlated with each other as well as total SRS scores (*r* > 0.80) and therefore biased the PLS results.

**Fig. 4:**
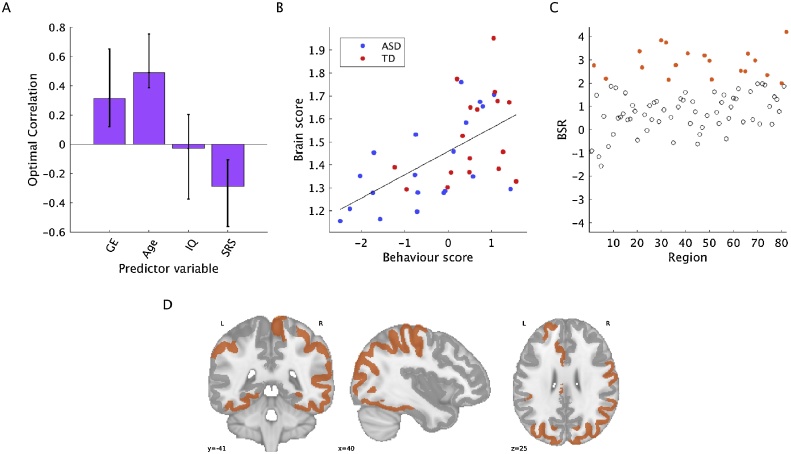
Behavioural PLS results for MSSD. A) Contrast in relationships for correlations between MSSD and predictor variables, B) associated brain and behaviour scores for each group, C) BSRs for each region, D) brain plot of BSRs. Regions with a BSR surpassing a threshold of +2 are shown in orange. Error bars show 95% confidence intervals determined through bootstrap resampling. Blue circles = ASD, red circles = TD.

**Fig. 5:**
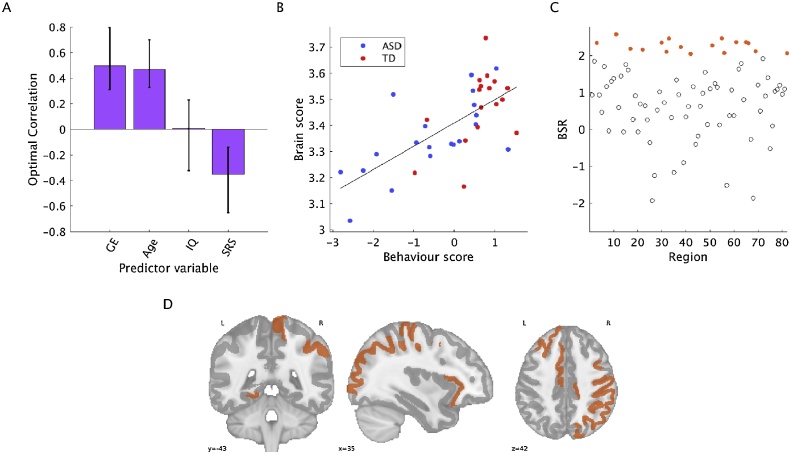
Behavioural PLS results for entropy. A) Contrast in relationships for correlations between entropy and predictor variables, B) associated brain and behaviour scores for each group, C) BSRs for each region, D) brain plot of BSRs. Regions with a BSR surpassing a threshold of +2 are shown in orange. Error bars show 95% confidence intervals determined through bootstrap resampling. Blue circles = ASD, red circles = TD.

The behavioral PLS analyses revealed one significant pattern for MSSD (*p* =  0.02, 58.11% covariance explained, Fig. 4) and for one significant pattern for entropy (*p* =  0.01, 48.88% covariance explained, Fig. 5). For both analyses, there was a distributed set of brain regions that exhibited positive correlations with GE and IQ, but negative correlations with SRS scores. For MSSD, 19 out of 82 (23.17% of) brain regions contributed reliably to the brain-behaviour pattern. For entropy, 17 out of 82 (20.73% of) brain regions contributed reliably to the pattern. These regions are listed in Supplementary Table 1. For both MSSD and entropy, a continuum of brain-behaviour relationships can be observed across ASD and TD participants, as shown in Figs. 4B and 5B. In other words, a broad range of brain and behaviour scores can be observed across all participants, but overall, brain and behaviour scores were higher in the TD group (MSSD brain scores: *t*(35) = −1.96, *p* =  0.06; MSSD behaviour scores: *t*(35) = −3.03, *p* =  0.005; entropy brain scores: *t*(35) = 2.40, *p* =  0.02; entropy behaviour scores: *t*(35) = 3.49, *p* =  0.001). Further, for both MSSD and entropy, behaviour scores were more variable for the ASD group compared to the TD group, which illustrates the highly heterogeneous nature of ASD, although the group difference did not reach significance for MSSD (MSSD, SD_ASD_ = 1.18, and SD_TD_ = 0.94, *F*(19, 16) = 2.29, *p* =  0.10; for entropy, SD_ASD_ = 1.23, SD_TD_ = 0.93, *F*(19, 16) = 3.64, *p* =  0.01).

Notably, as shown in Table 3, although the correlations between brain and behaviour scores were higher for the ASD group compared to the TD group, the correlations did not differ significantly between the diagnostic groups.

**Table 3:** Correlations between brain and behaviour scores.

Brain variable	All participants	ASD	TD	Significance
MSSD	*r(35)* = 0.58, *p* < 0.001	*r(18)* = 0.60, *p* = 0.005	*r(15)* = 0.38, *p* = 0.13	*Z* = 0.81, *p* = 0.42
Entropy	*r(35)* = 0.67, *p* < 0.001	*r(18)* = 0.67, *p* = 0.001	*r(15)* = 0.50, *p* = 0.04	*Z* = 0.72, *p* = 0.47

**Fig. 6:**
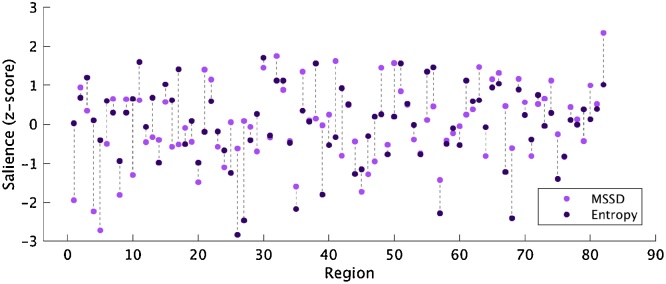
Brain saliences for MSSD and entropy. Light purple circles = MSSD, dark purple circles = entropy.

The brain saliences for the MSSD and entropy analyses were significantly correlated, *r* = 0.46, *p* <  0.001 (1000 permutations), showing that there was a strong relationship between the brain regions that contributed to the MSSD pattern and the regions that contributed to the entropy pattern (Fig. 6).

Behavioral PLS analysis was also performed in the ASD group using ADOS2 scores in addition to the other predictor variables. As ADOS2 scores were only available for the ASD group, the TD group was not included in these analyses. When ADOS2 total scores were included, the PLS analysis was significant for MSSD (*p* =  0.045), but not for entropy (*p* =  0.18). However, in both cases, the behaviour saliences were not reliable for ADOS2 total scores, as the 95% CIs crossed the x-axis. A similar pattern was observed when the ADOS2 social affect (SA) and RRB scores were used instead of the total scores: PLS was significant for MSSD (*p* =  0.02) and not significant for entropy (*p* =  0.15), but the behaviour saliences for ADOS2 SA and RRB scores were not reliable.

### 3.4 Interactions between MSSD-behaviour and entropy-behaviour correlations

As brain-behaviour relationships were similar for both MSSD and entropy, we combined both of these brain measures in a single behavioural PLS analysis, with each brain measure being treated as a separate “condition”, to determine if any additional patterns existed that showed a contrast between MSSD and entropy in terms of correlations with the predictor variables. However, the results of the analysis showed that there was one significant pattern (p < 0.001) showing the same pattern of brain-behaviour correlations as the individual analyses (i.e. positive correlations between brain variables and age and brain variables and GE, and negative correlations between brain variables and SRS scores). No additional significant patterns showing different brain-behaviour correlations for MSSD and entropy were observed.

### 3.5 Correlations between behaviour measures

**Fig. 7:**
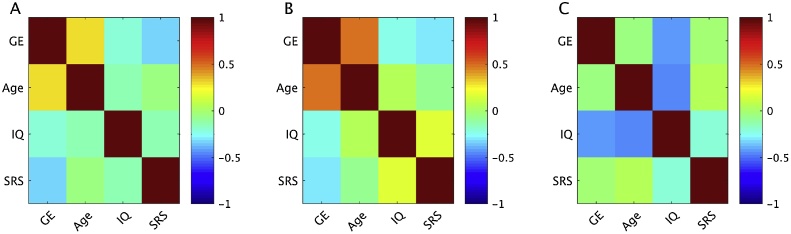
Correlation matrix showing relationships between the set of predictor variables used in the behavioral PLS for A) all participants, B) ASD participants, C) TD participants.

Finally, the relationships between the set of predictor variables were analyzed across all participants and within groups. As shown in Fig. 7, there was a moderate positive correlation between GE and age, a weak negative correlation between GE and IQ, and a moderate negative correlation between GE and SRS scores. SRS scores were weakly negatively correlated with IQ. Further, GE did not differ significantly between the ASD and TD groups, *t*(35) = −1.18, *p* =  0.25. A notable difference between groups was the correlation between age and GE: in ASD, this correlation was positive (*r(18)* = 0.46, *p* =  0.04), whereas the correlation was negative, but not significant, in controls (*r(15)* = -0.32, *p* =  0.21). The correlation coefficients between the two groups were significantly different, *Z* = 2.30, *p* =  0.02.

The correlation matrix for the predictor variables used in the ASD-only PLS analysis (i.e. including ADOS scores) is shown in Supplementary Fig. 4.

## 4 Discussion

### 4.1 Overview

Variability and complexity of resting-state BOLD signals were examined in participants with and without ASD. For both MSSD and entropy, a continuum of brain-behaviour relationships was observed across diagnostic groups despite a lack of significant differences between groups. A distributed set of brain regions exhibited positive correlations between MSSD and GE and age in both groups, and negative correlations with SRS scores. A similar pattern was observed for entropy.

### 4.2 Variability and complexity in ASD

Categorical and dimensional approaches were used to characterize MSSD and entropy in participants with ASD compared to TD participants. The categorical approach involved analyzing differences between diagnostic groups (ASD and controls), whereas the dimensional approach involved the use of continuous measures: age, IQ, SRS scores, and GE. No group differences were observed when a categorical approach was used; however, using the dimensional approach, a set of brain regions exhibited relationships with the predictor variables.

The negative relationship between entropy and SRS scores supports the notion that greater severity of ASD behaviours is associated with decreased entropy, and therefore decreased information processing capacity, in the brain. Few studies have examined entropy in ASD; however, our results are in line with a previous EEG study that found reduced MSE in ASD during both social and non-social tasks (Catarino et al., 2011). Reduced entropy in participants with more severe ASD behaviors supports the “loss of brain complexity hypothesis” proposed by Yang and Tsai (2013), which states that the complexity of neural signals reflects the capability of the brain to adapt to changing environments, and that in pathological states, this “adaptive capacity” of the brain is reduced.

Similarly, a continuum of brain-behaviour relationships was observed for MSSD, whereby SRS scores were negatively correlated with MSSD. A dimensional approach has been used to study MSSD in ADHD. Nomi et al. (2018) did not find significant differences in MSSD between children with and without ADHD, but found positive correlations between MSSD and ADHD behavioural severity across diagnostic categories. While Nomi et al. (2018) found positive correlations between MSSD and symptom severity, we found negative correlations between these measures. This finding may seem counterintuitive to theories suggesting that ASD is characterized by an increased ratio of excitatory-inhibitory coupling in the brain, which may lead to poor functional differentiation and noisier and unstable neural signaling, resulting in inefficient information processing (Rubenstein and Merzenich, 2003). One possible explanation for this discrepancy is that detrimentally increased levels of noise may manifest at smaller scales in ASD, but not at macroscopic scales as measured by fMRI. This hypothesis is in line with the theory that physical connectivity at the level of microcolumns is increased in ASD, but computational connectivity is reduced (Belmonte et al., 2004). In other words, within neural assemblies, connections between synapses and fiber tracts are increased, but long-range connections between functional brain regions are reduced. The authors hypothesized that increased physical connectivity could lead to undifferentiated neural regions, which would preclude the development of effective communication between long-range functional regions. Thus, at macroscopic scales, ASD-like symptoms may be associated with reduced variability, which could be associated with a reduced capacity to explore different functional network configurations. Further, we included a larger set of predictor variables in our study compared to Nomi et al.’s study. In addition to behavioral severity, we included age and a measure of information processing in structural networks (GE). This analysis revealed important interactions between GE, age, and behavioral severity, whereby for both MSSD and entropy, a distributed set of brain regions exhibited positive correlations with GE and age, but negative correlations with behavioral severity. Thus, factors such as the efficiency of structural networks and age may be modulating the relationship between MSSD, entropy and measures of behavioral severity.

The PLS analyses used in this study provided a data-driven, “systems” approach for understanding BOLD signal variability and complexity in ASD. For both MSSD and entropy, a set of distributed brain regions contributed reliably to the brain-behaviour patterns in the behavioural PLS analyses. Previous studies have also reported widespread alterations in measures of brain function in ASD. For instance, various studies have reported atypical FC in multiple resting-state networks (e.g. Abraham et al., 2017; Anderson et al., 2011; Rashid et al., 2018; Rudie et al., 2012; Supekar et al., 2013; Wee et al., 2016). Further, distributed abnormalities in white and grey matter development have been reported in ASD (Carper et al., 2002; Sparks et al., 2002). Müller (2007) suggested that ASD should be characterized as a “distributed disorder”, or in other words, a network disorder, which is applicable to genetics, brain structure and function, and behaviour. It was noted that the many potential genetic risk factors for ASD can influence the development of multiple networks in the brain; therefore, “localizing models” may not be helpful for understanding ASD. As stated by Müller (2007), it is likely that there is a “distributed ontogenetic starting point affecting several emerging brain regions and functional systems… and each of these will in turn affect additional regions and functional systems throughout development”. Therefore, data-driven, whole-brain analyses are important for understanding brain function in ASD.

### 4.3 Variability and entropy increase with age

Distributed brain regions showed increases in MSSD and entropy from childhood through adolescence, suggesting that across development, information processing capacity increases in the brain (Fig. 5, Fig. 6). Previous work has suggested that increased information processing capacity over development results from a greater quantity of possible configurations of functional networks (McIntosh et al., 2010). Entropy of EEG signals has been shown to increase from childhood to adulthood, and is associated with higher accuracy and less variable reaction times during task performance (Lippé et al., 2009; McIntosh et al., 2008; Misic et al., 2010). Increases in entropy across development are thought to reflect increased integration and segregation in the brain (Garrett et al., 2013; McIntosh et al., 2008).

While developmental changes in variability and entropy have mostly been studied using EEG, Nomi et al. (2017) used fMRI to study changes in variability across the lifespan. In this study, central executive, default mode, visual, sensorimotor, and subcortical regions exhibited linear decreases in MSSD from ages 6 to 85, whereas nodes in the salience network and bilateral ventral temporal cortices exhibited increases in variability. The discrepancies between these findings and ours may relate, in part, to differences in age ranges and preprocessing strategies, which can affect estimates of BOLD signal variability (Garrett et al., 2010, 2013). Additional research on developmental changes in BOLD signal variability across different age ranges using various preprocessing strategies is required to resolve these discrepancies.

### 4.4 Variability and entropy are associated with global efficiency in structural networks

Behavioural PLS also revealed positive correlations between MSSD of BOLD time series and GE in structural networks, and between entropy and GE (Fig. 5, Fig. 6). This finding supports the notion that structure shapes function in the brain (Deco et al., 2013; Honey et al., 2007, 2009, 2010). Further, there was a contrast in the relationship between GE and behavioural severity, such that brain regions that exhibited positive correlations with GE exhibited negative correlations with behavioural severity. Previous work has shown relationships between GE in structural networks and cognitive abilities. Berlot et al. (2016) showed that mild cognitive impairment (MCI) patients exhibited reduced GE of brain networks, which was associated with reduced cognitive control. Further, in Alzheimer’s disease, reduced GE has been associated with memory and executive function abilities (Reijmer et al., 2013). Collectively, these results suggest an important relationship information processing capacity in structural and functional networks and cognitive functioning.

### 4.5 Correlations between GE and age

While groups did not differ in GE, a significant positive correlation was found between GE and age in the ASD group, yet the correlation between GE and age in the TD group was negative and non-significant. A previous study of similarly-aged ASD and TD participants also did not find group differences in GE; however, they found that GE in structural networks increased with age (controlling for head motion) in TD, but not ASD, participants (Rudie et al., 2012). Another study reported a positive correlation between GE and age in TD individuals (Hagmann et al., 2010); however, the investigators studied a much broader age range (18 months to 18 years old). It is possible that developmental trajectories of GE are nonlinear, given that other properties of structural development do not follow linear trajectories. For instance, white matter integrity exhibits nonlinear increases from childhood to adulthood, with steeper increases in childhood (Lebel et al., 2008). Therefore, future studies should study larger samples and broader age ranges of individuals with and without ASD to examine potential nonlinearities and group differences in developmental trajectories of information processing capacity in structural networks. Another possibility is that efficiency trajectories may differ in different parts of the brain: Dennis et al. (2013) found that GE increased with age in the left hemisphere, but decreased with age in the right hemisphere, in a sample of 439 adolescents and adults aged 12 to 30 years. Thus, future studies should aim to further elucidate region- or network-dependent development of information processing capacity.

### 4.6 Limitations

There are several limitations of the current study. First, our sample size was small due to the limited number of high quality datasets with both DTI and resting-state fMRI data from the ABIDE II database. We chose to analyze data from a single site for this study. While previous work has demonstrated the feasibility of multisite DTI studies based on high concordance of fractional anisotropy and mean diffusivity measurements across five different scanning sites (Fox et al., 2012), the reliability of measurements of resting-state FC across scanning sites is less clear. FC measurements can be affected by differences in scanner manufacturers and acquisition protocols (Shinohara et al., 2017; Yu et al., 2018). Dansereau et al. (2017) analyzed multisite resting-state FC data across 8 scanning sites, and reported small to moderate between-site effects. Jovicich et al. (2016) studied difference in DMN FC in 5 healthy elderly participants who were scanned at 13 different sites with harmonized protocols, and found significant differences in temporal signal-to-noise ratio between sites. These differences were hypothesized to relate to differences in hardware and pulse sequences between sites. Nielsen et al. (2013) found that classification of ASD compared to controls based on resting-state FC was lower when multiple sites were included as opposed to a single site. Further, Easson et al. (2018) defined subtypes of ASD and controls based on FC using 5 sites from the ABIDE database, and found that it was necessary to regress out the effects of scan site prior to k-means classification, otherwise, the clusters differed significantly in scan site. This finding shows that prominent FC clusters defined in an unsupervised manner are driven by differences in FC across scanning sites. Thus, it will be crucial to further elucidate the effects of scanning site on resting-state FC to allow for multisite studies, and thus, larger sample sizes.

### 4.7 Conclusions

This study reveals relationships between brain signal variability and complexity and GE, age, and behavioural severity across ASD and TD participants, and illustrates the importance of taking a dimensional approach to studying brain function in ASD. By analyzing brain variability and complexity in relation to a set of predictor variables, a continuum of relationships between brain variables and predictor variables was observed; however, when treating ASD and TD groups categorically, significant group differences were not observed. Further, increased GE in structural networks and increased age were associated with higher MSSD and entropy, revealing important information about structure-function relationships in the brain and the developmental trajectories of variability and complexity.

## Conflict of interest

The authors declare no competing financial interests.

## References

[bib0005] Abraham A., Pedregosa F., Eickenberg M., Gervais P., Mueller A., Kossaifi J. (2014). Machine learning for neuroimaging with scikit-learn. Front. Neuroinform..

[bib0010] Abraham A., Milham M.P., Di Martino A., Craddock R.C., Samaras D., Thirion B., Varoquaux G. (2017). Deriving reproducible biomarkers from multi-site resting-state data: an Autism-based example. Neuroimage.

[bib0015] American Psychiatric Association (2013). Diagnostic and Statistical Manual of Mental Disorders.

[bib0020] Anderson J.S., Nielsen J.A., Froehlich A.L., DuBray M.B., Druzgal T.J., Cariello A.N. (2011). Functional connectivity magnetic resonance imaging classification of autism. Brain.

[bib0025] Belmonte M.K., Allen G., Beckel-Mitchener A., Boulanger L.M., Carper R.A., Webb S.J. (2004). Autism and abnormal development of brain connectivity. J. Neurosci..

[bib0030] Berlot R., Metzler-Baddeley C., Ikram M.A., Jones D.K., O’Sullivan M.J. (2016). Global efficiency of structural networks mediates cognitive control in mild cognitive impairment (2016). Front. Aging Neurosci..

[bib0035] Bezgin G., Vakorin V.A., van Opstal A.J., McIntosh A.R., Bakker R. (2012). Hundreds of brain maps in one atlas: registering coordinate-independent primate neuro-anatomical data to a standard brain. Neuroimage.

[bib0040] Bosl W., Tierney A., Tager-Flusberg H., Nelson C. (2011). EEG complexity as a biomarker for autism spectrum disorder risk. BMC Med..

[bib0045] Brett M., Hanke M., Cipollini B., Côté M., Markiewicz C., Gerhard S. (2016). nibabel: 2.1.0 (Version 2.1.0). Zenodo.

[bib0050] Bullmore E., Sporns O. (2012). The economy of brain network organization. Nat. Rev. Neurosci..

[bib0055] Campbell K., Grigg O., Saverino C., Churchill N., Grady C. (2013). Age differences in the intrinsic functional connectivity of default network subsystems. Front. Hum. Neurosci..

[bib0060] Carper R.A., Moses P., Tigue Z.D., Courchesne E. (2002). Cerebral lobes in autism: early hyperplasia and abnormal age effects. Neuroimage.

[bib0065] Catarino A., Churches O., Baron-Cohen S., Andrade A., Ring H. (2011). Atypical EEG complexity in autism spectrum conditions: a multiscale entropy analysis. Clin. Neurophysiol..

[bib0070] Chen H., Nomi J.S., Uddin L.Q., Duan X., Chen H. (2017). Intrinsic functional connectivity variance and state-specific under-connectivity in autism. Hum. Brain Mapp..

[bib0075] Churchill N.W., Strother S.C. (2013). PHYCAA+: an optimized, adaptive procedure for measuring and controlling physiological noise in BOLD fMRI. Neuroimage.

[bib0080] Churchill N.W., Oder A., Abdi H., Tam F., Lee W., Thomas C. (2012). Optimizing preprocessing and analysis pipelines for single-subject fMRI. I. Standard temporal motion and physiological noise correction methods. Hum. Brain Mapp..

[bib0085] Churchill N.W., Yourganov G., Oder A., Tam F., Graham S.J., Strother S.C. (2012). Optimizing preprocessing and analysis pipelines for single-subject fMRI: 2. Interactions with ICA, PCA, task contrast and inter-subject heterogeneity. PLoS One.

[bib0090] Churchill N.W., Spring R., Afshin-Pour B., Dong F., Strother S.C. (2015). An automated, adaptive framework for optimizing preprocessing pipelines in task-based functional MRI. PLoS One.

[bib0095] Ciric R., Wolf D.H., Power J.D., Roalf D.R., Baum G.L., Ruparel K. (2017). Benchmarking of participant-level confound regression strategies for the control of motion artifact in studies of functional connectivity. Neuroimage.

[bib0100] Constantino J.N., Gruber C.P. (2005). Social Responsiveness Scale (SRS).

[bib0105] Costa M., Goldberger A.L., Peng C.K. (2005). Multiscale entropy analysis of biological signals. Phys. Rev. E Stat. Nonlin. Soft Matter Phys..

[bib0110] Dansereau C., Benhajali Y., Risterucci C., Pich E.M., Orban P., Arnold D., Bellec P. (2017). Statistical power and prediction accuracy in multisite resting-state fMRI connectivity. Neuroimage.

[bib0115] Deco G., Jirsa V.K., McIntosh A.R. (2013). Resting brains never rest: computational insights into potential cognitive architectures. Trends Neurosci..

[bib0120] Dennis E.L., Jahanshad N., McMahon K.L., de Zubicaray G.I., Martin N.G., Hickie I.B. (2013). Development of brain structural connectivity between ages 12 and 30: a 4-Tesla diffusion imaging study in 439 adolescents and adults. Neuroimage.

[bib0125] Di Martino A., O’Connor D., Chen B., Alaerts K., Anderson J.S., Assaf M. (2017). Enhancing studies of the connectome in autism using the autism brain imaging data exchange II. Sci. Data.

[bib0130] Easson A.K., Fatima Z., McIntosh A.R. (2018). Functional connectivity-based subtypes of individuals with and without autism spectrum disorder. Netw. Neurosci..

[bib0135] Falahpour M., Thompson W.K., Abbott A.E., Jahedi A., Mulvey M.E., Datko M. (2016). Underconnected, but not broken? Dynamic functional connectivity MRI shows underconnectivity in autism is linked to increased intra-individual variability across time. Brain Connect..

[bib0140] Fox R.J., Sakaie K., Lee J.C., Debbins J.P., Liu Y., Arnold D.L. (2012). A validation study of multicenter diffusion tensor imaging: reliability of fractional anisotropy and diffusivity values. AJNR Am. J. Neuroradiol..

[bib0145] Fuchs E., Ayali A., Robinson A., Hulata E., Ben-Jacob E. (2007). Coemergence of regularity and complexity during neural network development. Dev. Neurobiol..

[bib0150] Garrett D.D., Kovacevic N., McIntosh A.R., Grady C.L. (2010). Blood oxygen level-dependent signal variability is more than just noise. J. Neurosci..

[bib0155] Garrett D.D., Kovacevic N., McIntosh A.R., Grady C.L. (2011). The importance of being variable. J. Neurosci..

[bib0160] Garrett D.D., Samanez-Larkin G.R., MacDonald S.W., Lindenberger U., McIntosh A.R., Grady C.L. (2013). Moment-to-moment brain signal variability: a next frontier in human brain mapping?. Neurosci. Biobehav. Rev..

[bib0165] Gatlin L. (1972). Information Theory and the Living System.

[bib0170] Ghosh A., Rho Y., McIntosh A.R., Kötter R., Jirsa V.K. (2008). Noise during rest enables the exploration of the brain’s dynamic repertoire. PLoS Comput. Biol..

[bib0175] Goldberger A.L., Peng C.K., Lipsitz L.A. (2002). What is physiologic complexity and how does it change with aging and disease?. Neurobiol. Aging.

[bib0180] Grady C.L., Garrett D.D. (2018). Brain signal variability is modulated as a function of internal and external demand in younger and older adults. Neuroimage.

[bib0185] Hagmann P., Sporns O., Madan N., Cammoun L., Pienaar R., Wedeen V.J. (2010). White matter maturation reshapes structural connectivity in the late developing human brain. Proc. Natl. Acad. Sci. U. S. A..

[bib0190] Heisz J.J., McIntosh A.R. (2013). Applications of EEG neuroimaging data: event-related potentials, spectral power, and multiscale entropy. J. Vis. Exp..

[bib0195] Heisz J.J., Shedden J.M., McIntosh A.R. (2012). Relating brain signal variability to knowledge representation. Neuroimage.

[bib0200] Honey C.J., Kötter R., Breakspear M., Sporns O. (2007). Network structure of cerebral cortex shapes functional connectivity on multiple time scales. Proc. Natl. Acad. Sci. U. S. A..

[bib0205] Honey C.J., Sporns O., Cammoun L., Gigandet X., Thiran J.P., Meuli R., Hagmann P. (2009). Predicting human resting-state functional connectivity from structural connectivity. Proc. Natl. Acad. Sci. U. S. A..

[bib0210] Honey C.J., Thivierge J.P., Sporns O. (2010). Can structure predict function in the human brain?. Neuroimage.

[bib0215] Hunter J.D. (2007). Matplotlib: A 2D graphics environment. Comput. Sci. Eng..

[bib0220] Jia H., Hu X., Deshpande G. (2014). Behavioral relevance of the dynamics of the functional brain connectome. Brain Connect..

[bib0225] Jovicich J., Minati L., Marizzoni M., Marchitelli R., Sala-Llonch R., Bartres-Faz D. (2016). Longitudinal reproducibility of default-mode network connectivity in healthy elderly participants: A multicentric resting-state fMRI study. Neuroimage.

[bib0230] Kötter R., Wanke E. (2005). Mapping brains without coordinates. Philos. Trans. R. Soc. Lond., B, Biol. Sci..

[bib0235] Lebel C., Walker L., Leemans A., Phillips L., Beaulieu C. (2008). Microstructural maturation of the human brain from childhood to adulthood. Neuroimage.

[bib0240] Lippé S., Kovacevic N., McIntosh A.R. (2009). Differential maturation of brain signal complexity in the human auditory and visual system. Front. Hum. Neurosci..

[bib0245] McIntosh A.R., Lobaugh N.J. (2004). Partial least squares analysis of neuroimaging data: applications and advances. Neuroimage.

[bib0250] McIntosh A.R., Bookstein F.L., Haxby J.V., Grady C.L. (1996). Spatial pattern analysis of functional brain images using partial least squares. Neuroimage.

[bib0255] McIntosh A.R., Kovacevic N., Itier R.J. (2008). Increased brain signal variability accompanies lower behavioral variability in development. PLoS Comput. Biol..

[bib0260] McIntosh A.R., Kovacevic N., Lippé S., Garrett D., Grady C., Jirsa V. (2010). The development of a noisy brain. Arch. Ital. Biol..

[bib0265] Misic B., Mills T., Taylor M.J., McIntosh A.R. (2010). Brain noise is task dependent and region specific. J. Neurophysiol..

[bib0270] Müller R.A. (2007). The study of autism as a distributed disorder. Mental Retardation Dev. Disabilities Res. Rev..

[bib0275] Nelson S.B., Valakh V. (2015). Excitatory/inhibitory balance and circuit homeostasis in autism spectrum disorders. Neuron.

[bib0280] Nielsen J.A., Zielinski B.A., Fletcher P.T., Alexander A.L., Lange N., Bigler E.D., Lainhart J.E., Anderson J.S. (2013). Multisite functional connectivity MRI classification of autism: ABIDE results. Front. Hum. Neurosci..

[bib0285] Nomi J.S., Bolt T.S., Ezie C.E.C., Uddin L.Q., Heller A.S. (2017). Moment-to-moment BOLD signal variability reflects regional changes in neural flexibility across the lifespan. J. Neurosci..

[bib0290] Nomi J.S., Schettini E., Voorhies W., Bolt T.S., Heller A.S., Uddin L.Q. (2018). Resting-state brain signal variability in prefrontal cortex is associated with ADHD symptom severity in children. Front. Hum. Neurosci..

[bib0295] Pincus S.M. (1991). Approximate entropy as a measure of system complexity. Proc. Natl. Acad. Sci. U. S. A..

[bib0300] Pruim R.H.R., Mennes M., Buitelaar J.K., Beckmann C.F. (2015). Evaluation of ICA-AROMA and alternative strategies for motion artifact removal in resting state fMRI. Neuroimage.

[bib0305] Pruim R.H.R., Mennes M., van Rooij D., Llera A., Buitelaar J.K., Beckmann C.F. (2015). ICA-AROMA: a robust ICA-based strategy for removing motion artifacts from fMRI data. Neuroimage.

[bib0310] Rashid B., Blanken L.M.E., Muetzel R.L., Miller R., Damaraju E., Arbabshirani M.R. (2018). Connectivity dynamics in typical development and its relationship to autistic traits and autism spectrum disorder. Hum. Brain Mapp..

[bib0315] Reijmer Y.D., Leemans A., Caeyenberghs K., Heringa S.M., Koek H.L., Biessels G.J., Utrecht Vascular Cognitive Impairment Study Group (2013). Neurology.

[bib0320] Richman J.S., Moorman J.R. (2000). Physiological time-series analysis using approximate entropy and sample entropy. Am. J. Physiol. Heart Circ. Physiol..

[bib0325] Rubenstein J.L., Merzenich M.M. (2003). Model of autism: increased ratio of excitation/inhibition in key neural systems. Genes Brain Behav..

[bib0330] Rubinov M., Sporns O. (2010). Complex network measures of brain connectivity: uses and interpretations. Neuroimage.

[bib0335] Rudie J.D., Brown J.A., Beck-Pancer D., Hernandez L.M., Dennis E.L., Thompson P.M. (2012). Altered functional and structural brain network organization in autism. Neuroimage Clin..

[bib0340] Sanz Leon P., Knock S.A., Woodman M.M., Domide L., Mersmann J., McIntosh A.R., Jirsa V. (2013). The Virtual Brain: a simulator of primate brain network dynamics. Front. Neuroinform..

[bib0345] Shannon C.E. (1948). A mathematical theory of communication. Bell Syst. Tech. J..

[bib0350] Shinohara R.T., Oh J., Nair G., Calabresi P.A., Davatzikos C., Doshi J. (2017). Volumetric analysis from a harmonized multisite brain MRI study of a single subject with multiple sclerosis. AJNR Am. J. Neuroradiol..

[bib0355] Smith R.E., Tournier J.D., Calamante F., Connelly A. (2012). Anatomically-constrained tractography: improved diffusion MRI streamlines tractography through effective use of anatomical information. Neuroimage.

[bib0360] Sparks B.F., Friedman S.D., Shaw D.W., Aylward E.H., Echelard D., Artru A.A. (2002). Brain structural abnormalities in young children with autism spectrum disorder. Neurology.

[bib0365] Stephan K.E., Kamper L., Bozkurt A., Burns G.A., Young M.P., Kötter R. (2001). Advanced database methodology for the Collation of Connectivity data on the Macaque brain (CoCoMac). Philos. Trans. R. Soc. Lond., B, Biol. Sci..

[bib0370] Supekar K., Uddin L.Q., Khouzam A., Phillips J., Gaillard W.D., Kenworthy L.E. (2013). Brain hyperconnectivity in children with autism and its links to social deficits. Cell Rep..

[bib0375] Van Essen D.C., Dierker D.L. (2007). Surface-based and probabilistic atlases of primate cerebral cortex. Neuron.

[bib0380] Van Essen D.C., Drury H.A., Dickson J., Harwell J., Hanlon D., Anderson C.H. (2001). An integrated software suite for surface-based analyses of cerebral cortex. J. Am. Med. Inform. Assoc..

[bib0385] von Neumann J., Kent R.H., Bellison H.R., Hart B.I. (1941). The mean square successive difference. Ann. Math. Stat..

[bib0390] Wee C.Y., Yap P.T., Shen D. (2016). Diagnosis of autism Spectrum disorders using temporally distinct resting-state functional connectivity networks. CNS Neurosci. Ther..

[bib0395] Yang A.C., Tsai S.J. (2013). Is mental illness complex? From behavior to brain. Prog. Neuropsychopharmacol. Biol. Psychiatry.

[bib0400] Yang A.C., Huang C.C., Yeh H.L., Liu M.E., Hong C.J., Tu P.C., Tsai S.J. (2013). Complexity of spontaneous BOLD activity in default mode network is correlated with cognitive function in normal male elderly: a multiscale entropy analysis. Neurobiol. Aging.

[bib0405] Yang A.C., Tsai S.J., Lin C.P., Peng C.K. (2018). A strategy to reduce bias of entropy estimates in resting-state fMRI signals. Front. Neurosci..

[bib0410] Yu M., Linn K.A., Cook P.A., Phillips M.L., McInnis M., Fava M. (2018). Statistical harmonization corrects site effects in functional connectivity measurements from multi-site fMRI data. Hum. Brain Mapp..

[bib0415] Zhang J., Cheng W., Liu Z., Zhang K., Lei X., Yao Y. (2016). Neural, electrophysiological and anatomical basis of brain-network variability and its characteristic changes in mental disorders. Brain.

