# Supplementary material for: BOLD signal variability and complexity in children and adolescents with and without autism spectrum disorder
Source: Dev Cogn Neurosci. 2019 Mar 5;36:100630. doi: 10.1016/j.dcn.2019.100630 (PMC6969202; doi:10.1016/j.dcn.2019.100630)
Supplement: Supplementary file 1 [file mmc1.docx]

**Supplementary Material**

**Effects of motion censoring on MSSD and entropy**

PLS analyses were performed on MSSD and sample entropy estimates using two “conditions”: 1) the original preprocessing steps, 2) the original preprocessing steps excluding motion censoring. Not surprisingly, there were several regions that exhibited higher variability when censoring was not used (p = 0.03; Supplementary Figure 1). Censoring did not significantly affect entropy estimates; the PLS analysis for entropy comparing these two sets of preprocessing steps was not significant (p = 0.15).


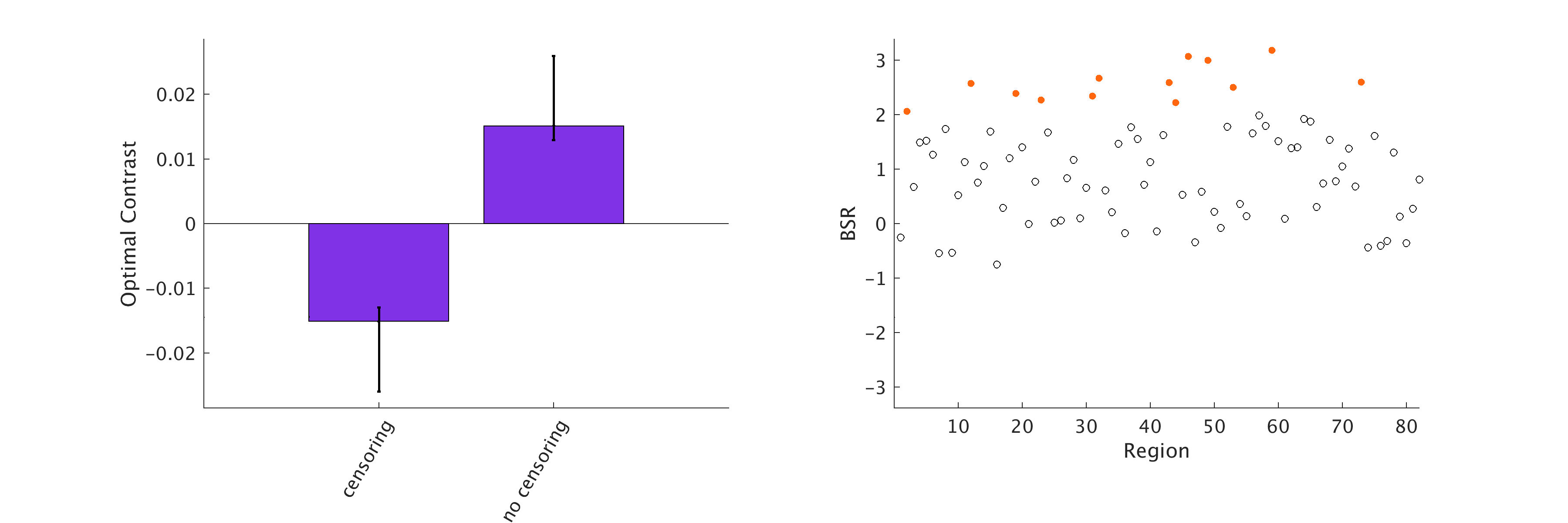


**Supplementary Figure 1.** Contrast in MSSD estimates between censoring conditions and associated BSRs for each region, at a threshold of +2. Error bars show 95% confidence intervals determined through bootstrap resampling.

The use of censoring did not impact the overall findings of the study: when censoring was not used, there were no categorical group differences for MSSD (p = 0.18) or entropy (p = 0.19), and similar brain-behaviour relationships were found for MSSD (p = 0.01, 58.10% covariance explained; Supplementary Figure 2) and entropy (p = 0.01, 49.86% covariance explained; Supplementary Figure 3), where there were positive correlations between brain variables and age and brain variables and GE, and negative correlations between brain variables and SRS scores.


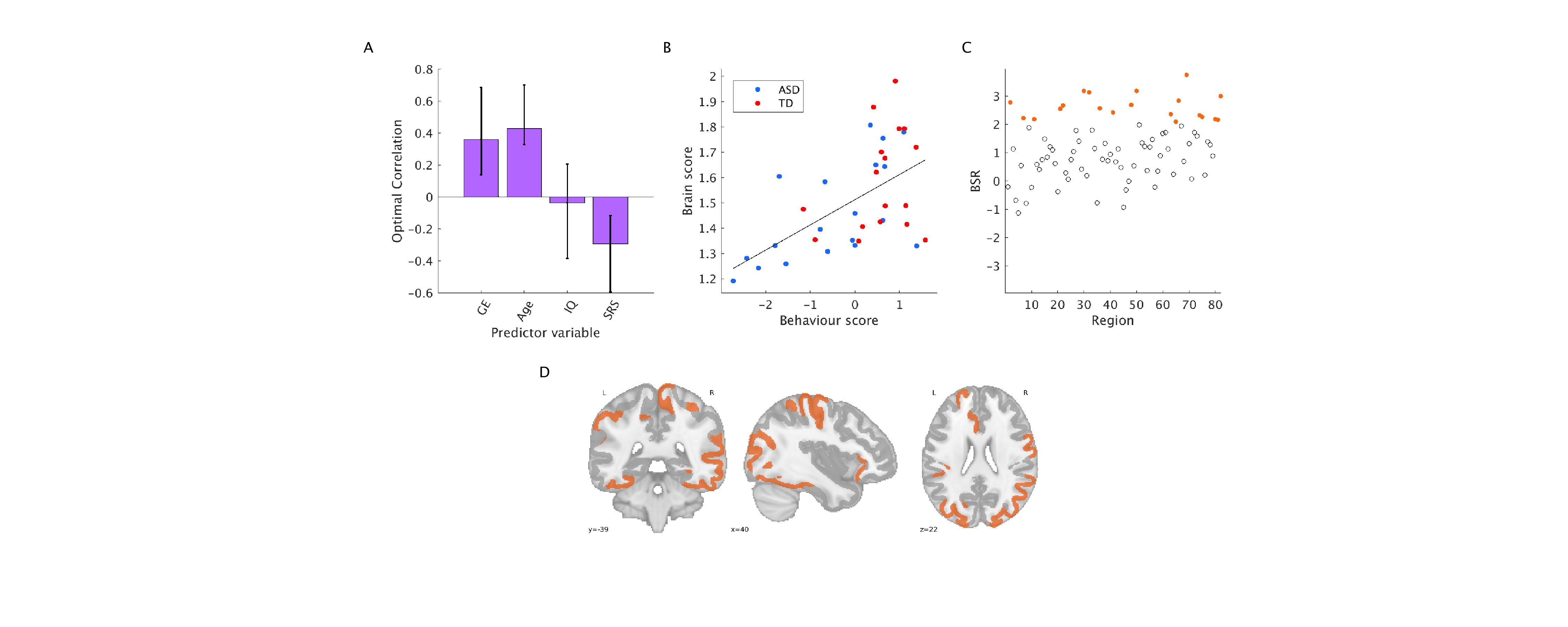


**Supplementary Figure 2.** Behavioural PLS results for MSSD, when motion censoring was not used as a preprocessing step. Regions with a BSR surpassing a threshold of +2 are shown in orange. Error bars show 95% confidence intervals determined through bootstrap resampling. Blue circles = ASD, red circles = TD.


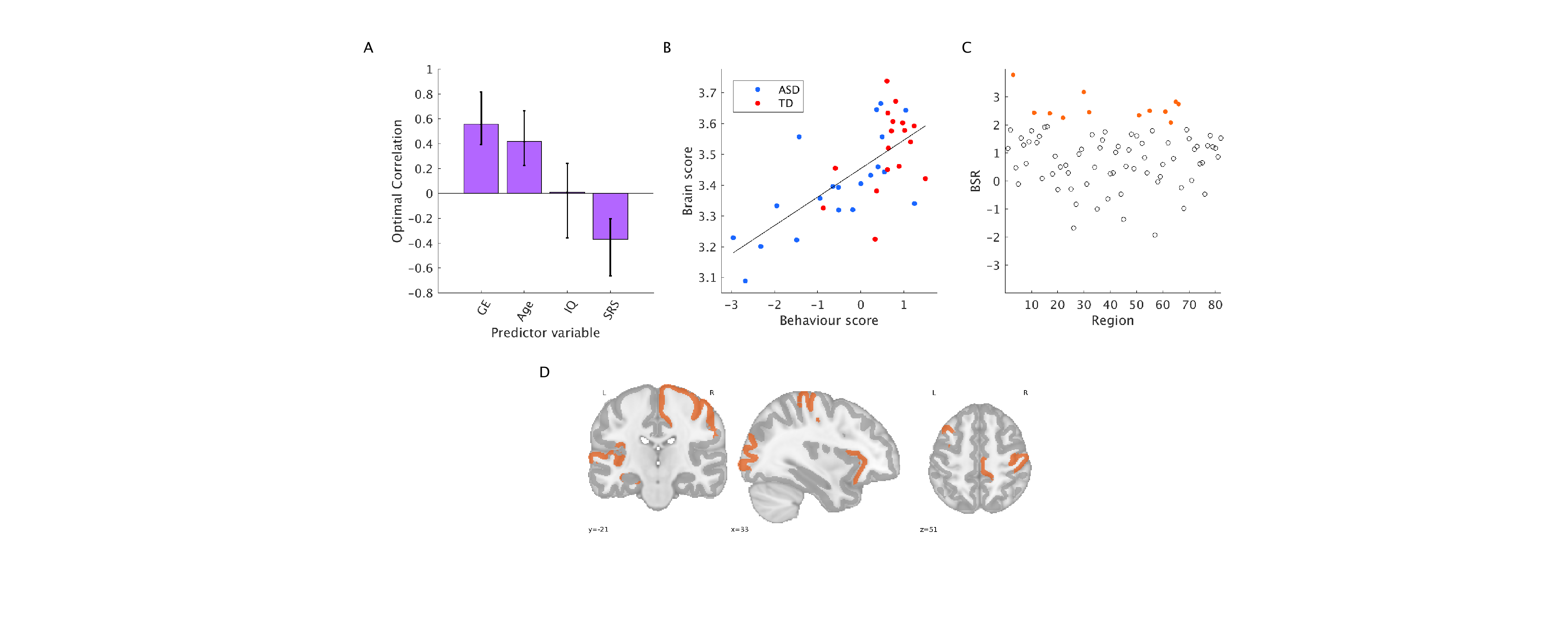


**Supplementary Figure 3.** Behavioural PLS results for entropy, when motion censoring was not used as a preprocessing step. A) Contrast in relationships for correlations between entropy and predictor variables, B) associated brain and behaviour scores for each group, C) BSRs for each region, D) brain plot of BSRs. Regions with a BSR surpassing a threshold of +2 are shown in orange. Error bars show 95% confidence intervals determined through bootstrap resampling. Blue circles = ASD, red circles = TD.

**Supplementary Table 1:** Brain regions exceeding a BSR threshold of + 2 in the behavioural PLS analyses

| **MSSD** | **Entropy** |
| --- | --- |
| Right superior temporal cortex  Right central temporal cortex  Right ventral temporal cortex  Right anterior visual area, dorsal part  Right primary somatosensory cortex  Right primary motor cortex  Right inferior parietal cortex  Right intraparietal cortex  Right dorsolateral premotor cortex  Left central temporal cortex  Left inferior temporal cortex  Left parahippocampal cortex  Left anterior visual area, dorsal part  Left centrolateral prefrontal cortex  Left secondary auditory cortex  Left anterior cingulate cortex  Left inferior parietal cortex  Left dorsolateral prefrontal cortex  Left dorsolateral premotor cortex | Right amygdala  Right gustatory cortex  Right subgenual cingulate cortex  Right anterior visual area, dorsal part  Right primary somatosensory cortex  Right primary motor cortex  Right inferior parietal cortex  Right frontal eye field  Left temporal polar cortex  Left parahippocampal cortex  Left posterior insula  Left prefrontal polar cortex  Left medial prefrontal cortex  Left centrolateral prefrontal cortex  Left secondary auditory cortex  Left anterior cingulate cortex  Left dorsolateral premotor cortex |


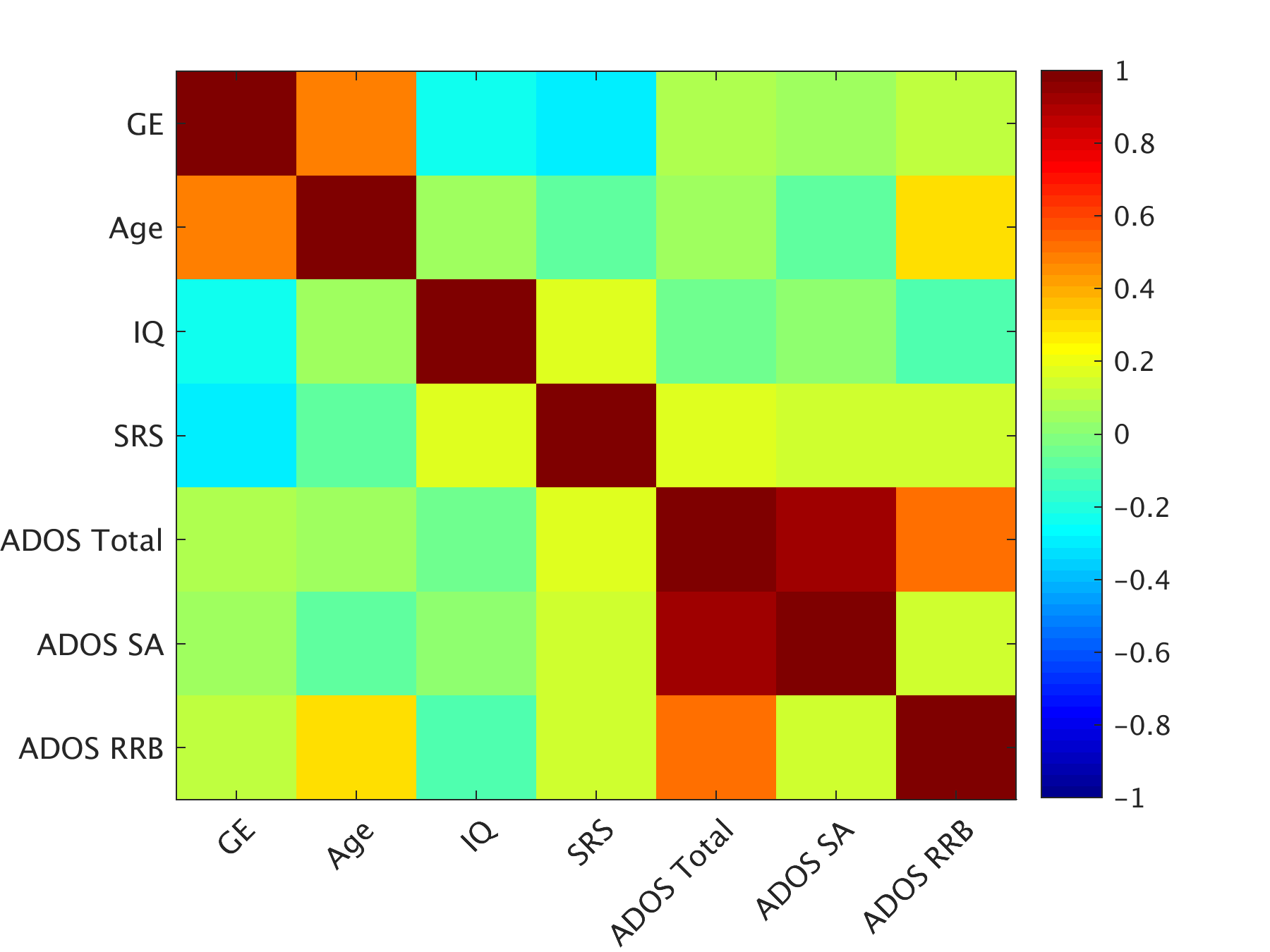


**Supplementary Figure 4.** Correlation matrix for the set of behaviour variables used in the behavioral PLS analysis for the ASD group.
